# How do we best engage young people in decision-making about their health? A scoping review of deliberative priority setting methods

**DOI:** 10.1186/s12939-022-01794-2

**Published:** 2023-01-25

**Authors:** Daniella Watson, Mimi Mhlaba, Gontse Molelekeng, Thulani Andrew Chauke, Sara Correia Simao, Sarah Jenner, Lisa J. Ware, Mary Barker

**Affiliations:** 1 Global Health Research Institute, Human Development and Health, Faculty of Medicine, University of Southampton, Southampton, UK; 2 SAMRC Developmental Pathways for Health Research Unit, School of Clinical Medicine, University of the Witwatersrand, Johannesburg, South Africa; 3 Department of Global Health and Social Medicine, King’s College London, London, UK; 4 Department of Adult, College of Education, Community and Continuing Education, University of South Africa, Pretoria, South Africa; 5 Medical Research Council Lifecourse Epidemiology Centre, University of Southampton, Southampton, UK; 6 DSI-NRF Centre of Excellence in Human Development, University of the Witwatersrand, Johannesburg, South Africa; 7 School of Health Sciences, Faculty of Life and Environmental Sciences, University of Southampton, Southampton, UK; 8 School of Public Health, Faculty of Health Sciences, University of the Witwatersrand, Johannesburg, South Africa; 9 NIHR Southampton Biomedical Research Centre, University Hospitals Southampton NHS Foundation Trust, Southampton, UK

**Keywords:** Young people, Adolescents, Priority setting, Scoping review, Health decisions

## Abstract

**Introduction:**

International organisations have called to increase young people’s involvement in healthcare and health policy development. We currently lack effective methods for facilitating meaningful engagement by young people in health-related decision-making. The purpose of this scoping review is to identify deliberative priority setting methods and explore the effectiveness of these in engaging young people in healthcare and health policy decision-making.

**Methods:**

Seven databases were searched systematically, using MeSH and free text terms, for articles published in English before July 2021 that described the use of deliberative priority setting methods for health decision-making with young people. All titles, abstracts and full-text papers were screened by a team of six independent reviewers between them. Data extraction followed the Centre for Reviews and Dissemination guidelines. The results are presented as a narrative synthesis, structured around four components for evaluating deliberative processes: 1) representation and inclusion of diverse participants, 2) the way the process is run including levels and timing of participant engagement, 3) the quality of the information provided to participants and 4) resulting outcomes and decisions.

**Findings:**

The search yielded 9 reviews and 21 studies. The more engaging deliberative priority setting tools involved young people-led committees, mixed methods for identifying and prioritising issues and digital data collection and communication tools. Long-term and frequent contact with young people to build trust underpinned the success of some of the tools, as did offering incentives for taking part and skills development using creative methods. The review also suggests that successful priority setting processes with young people involve consideration of power dynamics, since young people’s decisions are likely to be made together with family members, health professionals and academics.

**Discussion:**

Young people’s engagement in decision-making about their health is best achieved through investing time in building strong relationships and ensuring young people are appropriately rewarded for their time and contribution. If young people are to be instrumental in improving their health and architects of their own futures, decision-making processes need to respect young people’s autonomy and agency. Our review suggests that methods of power-sharing with young people do exist but that they have yet to be adopted by organisations and global institutions setting global health policy.

**Supplementary Information:**

The online version contains supplementary material available at 10.1186/s12939-022-01794-2.

## Introduction

Organisations including the World Health Organisation (WHO), the United Nations International Children’s Emergency Fund (UNICEF), the Wellcome Trust and the Lancet recognise the importance of engaging young people in decision-making about their health [1]. The COVID-19 pandemic has had disproportionate impact on the lives and health of young people around the world [2]. The WHO–UNICEF–Lancet Commission on ‘a future for the world’s children?’ has launched a call for involvement of young people in all decision-making policies and coalitions [3, 4].

Young people make up approximately a quarter of the world’s population [5, 6]. Investing in young people’s health has the “triple benefit” of improving their current health, their health as adults and the health of their children [7]. Adolescence is a critical period of the lifecourse, during which young people undergo the physical and psychological transitions that accompany pubertal growth in early adolescence (10–14 years), brain maturation, and social and emotional development in later adolescence (15–19 years) [8]. They start to gain autonomy over life decisions, depending less on their parents and becoming more susceptible to the influence of their peers. They make important health and social choices that often persist into adulthood [9–11]. These powerful physical and psychological changes represent a unique phase between childhood and adulthood, which requires specific consideration and services. The Association of Young People’s Health emphasise the need to conceptualise how health inequalities arise in young people, which they position as underpinned by the young person’s economic inequality which shapes their social determinants [12]. This leads to variability in how the young people access and experience services and support, their health behaviours and relationships with parents, carers and peers. This will ultimately shape their physical and mental health [12].

Over 30 years ago, the United Nations Convention on the Rights of the Child determined the right of young people to make their own life decisions, taking into account their age and maturity [13]. In her annual report for 2021, the UN youth envoy urges international organisations and institutions to acknowledge this commitment and to make decisions based on working with and listening to young people in a meaningful way [14]. Tools such as those developed by Save the Children and Women Deliver are excellent means of supporting young people to develop skills in advocacy [15, 16]. We still lack, however, effective methods for facilitating meaningful engagement of young people in setting priorities for health policy and healthcare decisions that affect their lives, livelihoods and futures.

There are a number of recognised priority setting techniques used most commonly with adults [17]. These tend to be categorised by two levels of engagement. The first is a non-deliberative approach which consults members of the public about their priorities, final decisions about which are made by those running the process [18–25]. Focus groups and interviews tend not to facilitate discussions about trade-offs in decision-making, such that having more of one option potentially results in having less of another, which is a key feature in priority setting [17]. The second is a deliberative approach which engages members of the public in a two-way dialogue and results in a set of priorities agreed with healthcare providers, policy makers and health researchers. Deliberative approaches have value over non-deliberative as they involve a process that brings together different points of view to derive a consensus without coercion, deception or manipulation [26]. This is both a more equitable and respectful way to involve the public in health policy and healthcare decisions and is likely to create more effective health services as they deliver what people prioritise. Deliberations also facilitate discussions about trade-offs and expectations when setting priorities.

Manafo and colleagues conducted a scoping review of public and patient priority setting, and identified five successful deliberative methods [17]: James Lind Alliance Priority Setting partnership (United Kingdom) [14], Dialogue methods (Netherlands) [16], Deep Inclusion (United States) [17], Choosing All Together (United States) [18], and Global Evidence Mapping (Australia and New Zealand) [27]. To date, these deliberative methods have rarely been used with young people to engage them in setting priorities for their health care. It has been more usual to use non-deliberative methods in consultations with young people [28–36]. The purpose of this scoping review is to identify and evaluate deliberative priority setting methods that have been used to engage young people in healthcare and health policy decisions.

### Research questions

1. Which deliberative methods have been used to engage young people in healthcare and health policy priority setting?
2. What features of these methods make them effective in engaging young people with healthcare and health policy priority setting?

## Methods

### Study selection

A scoping review was conducted in order to identify and evaluate deliberative priority setting tools that have been used to engage young people with healthcare and health policy decisions. This review method was chosen in order to produce a rapid account of the extent, range and nature of key tools [37]. We searched Prospero for existing reviews of priority setting in healthcare with young people, and as none were found we progressed with designing the scoping review strategy. The initial search was conducted in May 2019 and included major medical and social science databases including Cochrane library, Embase, MEDLINE, psychINFO, web of science, and CINAHL. There was no restriction on the publication date, and a complete search strategy can be found in Additional file 1. The search was updated in July 2021. The search strategy was narrow in order to focus on priority setting in healthcare with young people in particular. We engaged via meetings and emails with UNICEF experts and academic experts in adolescent health and priority setting known to the research team, to identify papers missing from the database search.

Papers were stored using Endnote version X9 and duplicates were removed. The papers were then screened using the Rayyan QCRI app and website [38]. All titles and abstracts were screened by one reviewer, and a second reviewer from the review team screened 10%. The full-text of relevant papers was obtained and assessed against the inclusion criteria (see Table 1). Studies were included that used a mixed-methods design including literature reviews, qualitative and quantitative methods, involved young people aged 10–24 years [39] and were based on priority setting to determine healthcare decisions. Bibliographies of retrieved papers were searched and experts in young people priority setting identified an additional paper. Healthcare decisions encompassed both medical and dental decisions. Exclusion of papers that did not meet the inclusion criteria was agreed by the review team.

**Table 1:** Inclusion and exclusion criteria

	Inclusion	Exclusion
**Study design**	Interventional studies or evaluation of interventions studies. Experimental and quasi-experimental studies.	Observational studies.
**Population**	Any population/group of a young people, age between 10 and 24 years.	Children below age of 10 years. Adult populations above age 24 years.
**Intervention**	Interventions explicitly using deliberative priority setting methods.	Interventions using non-deliberative priority setting methods such as solely focus groups, interviews and surveys.
**Outcome**	Decision-making and achievement of consensus by young peoples.	Decision-making and achievement of consensus solely by parents, caregivers, health professionals, educators, or other adult stakeholders.

### Data analysis

Papers were screened using the following definition of deliberative priority setting:

1. Research planning included gathering and analysing identified research priorities by engaging patients and the public along with clinicians and researchers.
2. Followed by prioritization of topics through dialogue between all stakeholders [26]

**Fig. 1:**
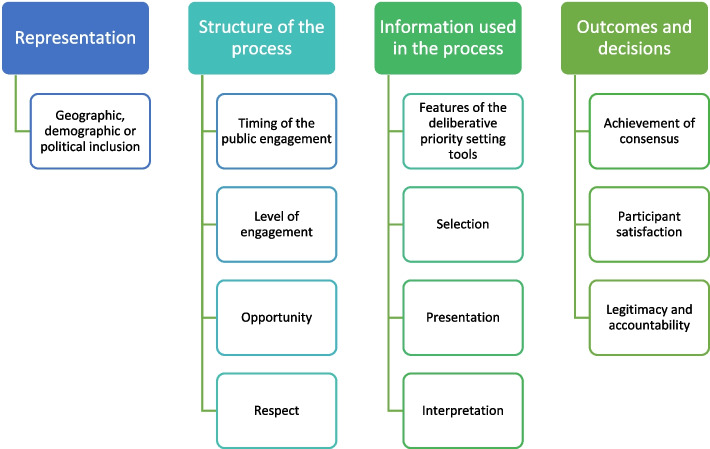
Flow chart of key components for evaluating deliberative processes. Adopted from Abelson et al. 2003

To focus the data analysis, we used Abelson et al’s four components for evaluating deliberative processes: 1) representation, referring to geographic, demographic or political inclusion of young people; 2) the structure of the process or procedures, emphasising the timing of public engagement in decision-making processes, the level of engagement, and opportunity to share views and gain mutual respect; 3) the quality of information given to participants in terms of how it is selected, presented and interpreted for them; and 4) the outcomes and decisions arising from the process, in terms of achievement of consensus, participant satisfaction, and legitimacy and accountability (see Fig. 1) [40]. As the current review aims to identify and describe different types of deliberative methods that have been used to engage young people, we first outline the features of each deliberative priority setting tool before evaluating them using Abelson’s evaluation criteria.

### Data synthesis

A summary table was produced (Table 2) which describes the characteristics of the identified studies including study design, priority setting features, setting and participants, assessment measures and conclusions. The summary table was used as the basis of a narrative synthesis of data examining and evaluating the types and effectiveness of deliberative priority setting techniques used to engage young people with healthcare and health policy decisions. Assessing the quality of included studies is unnecessary in a scoping review because it allows for a greater range of study designs and methodologies than a systematic review [37].

**Table 2:** Description of included reviews and studies

#	Author, year	Study design	Priority setting features	Setting and participants	Measurements	Discussion
	Review articles (*n* = 9)					
1	Boland et al. 2019 [41]	Systematic review to identify barriers to and facilitators of SDM	Innovation was SDM or a collaborative decision-making approach between HCPs, parents, and/or children;	Paediatric Clinical Practice Participants were children or young people up to age 18, or carers of the chid or young person. HCPs (*n* = 19), parents (*n* = 18), children (*n* = 8), multiple perspectives (*n* = 26), and observers (*n* = 7)	Ottawa Model of Research Use measured: • Decision Level • Innovation level • Adopters level • Relational level • Practice environment	• The majority of the SDM approaches were conducted on both parents and were found to be successful at decision making. • Power imbalances influenced the magnitude of how much the young people’s views informed the final decision. • HCPs and family made for difficult decision-making negotiations • Capacity building of HCP to support families with high quality informatio***n***
2	Cheng et al., 2017, United Kingdom, [42]	Scoping review to identify SDM approaches	Search strategy was developed to capture SDM approaches.	Participants were children or young people up to age 18, or carers of the chid or adolescent. The setting was mental health services. 12 reviews aimed at parents, 8 reviews aimed at children and adolescents, and 2 aimed at both young people and parents.	22 papers were included, and grouped into six approaches: • therapeutic techniques, • psychoeducational information • decision aids • action planning or goal setting • discussion prompts • mobilizing patients to engage	• The majority of the SDM approaches were conducted with parents and young person, and were found to be successful at decisions making. • However solely young people’s decision making was barely discussed.
3	Feenstra et al., 2014, Canada [43]	Systematic review of effective interventions that support decision making of children	Search strategy was developed to include decision making as an outcome, solely for youth and child health.	Young people under 18 years who are facing a health issue and seeking health services for risky behaviours, learning difficulties and end of life planning. All studies were conducted in the United States.	The priority setting techniques included decision coaching, coaching plus educational aid, and education alone.	• Five studies in six papers were identified. • Lack of interventions focused on supporting young people’s decision making. • Studies included evaluation of parent and child decision but did not discuss children’s capacity to make their own decisions.
4	Gurung et al. 2020 [44]	Systematic scoping review on public engagement strategies in the paediatric health-care setting.	The experiences/outcomes/impact of patient, family and public engagement strategies in paediatric services.	Focused on patient, family and public engagement strategies in the paediatric health-care setting in high-income countries.	Most studies we identified examined child/youth/parent satisfaction with care after the implementation of an engagement strategy.	• 21 identified • Absence of studies reviewing strategies targeting engagement with children/youth and families at the ‘service design and strategy’ and ‘policy-making’ levels. • Fewer examined whether the implementation of the strategy produced changes in treatment outcomes and service improvement.
5	Malone et al., 2019 [45]	Systematic review to assess the effectiveness of interventions that promote participation in shared decision-making for children and young people	To promote SDM for children and adolescents with cystic fibrosis supported by parents or at healthcare professionals	Children and adolescents diagnosed with cystic fibrosis aged between four and 18 years. SDM with parents, carers and any healthcare professionals (e.g. doctors, nurses, allied professionals).	The final 26 studies that were reviewed were excluded, with reasons being mainly due to being non-RTC or the intervention not being appropriate.	• Authors did not find any studies that were eligible to include in the review. • Recommendation for future research to aim to test models of SDM for young people with CF, which could be developed from existing models for adults; and also work out which methods young people prefer.
6	Pyke-Grimm et al., 2019 [46]	Young people’s involvement in cancer treatment decision making.	Young people’s involvement in their treatment decision making and within the context of their family and with their HCPs	Young people aged 15– 21 years. Also researched for studies including parents and health care providers of young people with cancer.	Influencing factors: • Young people’s preferred, actual, and perceived involvement • age and cognitive maturity, disease and illness factors, information and communication • relationships, roles, and perspectives of parents and healthcare providers.	• 21 articles identified • Decisions were influenced by the magnitude and timing of the decision, chronological and developmental stage, diseases stage, previous experience with diseases type and magnitude of the decision, and decisional and family context. • Young people’s role in decision making is situational and often evolved with time.
7	Wijngaarde et al., 2021 [47]	Scoping review to explore SDM interventions and their effectiveness in terms of participation, knowledge, decisional conflict, satisfaction health-related quality of life, and treatment adherence for chronically and critically ill children	Addressed child participation in treatment decision-making, knowledge, decisional conflict, health-related quality of life, and treatment adherence	Researchers searched relevant medical databases published between January 2008 and January 2020 for studies targeting children aged 4–18 years old, suffering from a chronic and/or critical disease.	SDM interventions mostly used were decision aids (*n* = 8), questionnaires for caretakers/parents and children (*n* = 4), and a SDM toolkit (n = 2). Patient decision aids were used in all studies. Data from the outcomes included a child’s level of participation, number of participants, tools for participation, and the use of eHealth tools in pediatric SDM.	• Nine studies identified. • Study shows growing awareness amongst clinicians and parents that a child’s participation in SDM can lead to better health outcomes and improved knowledge about the disease and treatment options. • Better knowledge decreases decisional conflict while positively impacting treatment adherence.
8	Wyatt et al., 2015 [48]	Systematic review and meta-analysis on interventions engaging paediatric and parent decision-making	Meta-analysis was performed on three outcomes: knowledge, decisional conflict, and satisfaction of the shared decision making process.	The majority (*n* = 34) of interventions targeted parents alone, a minority targeted the paediatric patient alone (*n* = 4) and clinician alone (*n* = 3). Other studies targeted more than one stakeholder (*n* = 14), with the most frequently targeted the patient and parent dyad (*n* = 6).	Knowledge was assessed as percentage of questions correctly answered. Decisional conflict was assessed by feeling uninformed, feeling unclear about personal values, feeling unsupported in decision making. Satisfaction measured using a variety of non-standardized scales.	• 54 studies identified 15 studies were reported the in meta-analysis • Meta-analysis revealed SDM interventions significantly improved knowledge, reduced decisional conflict, but increased satisfaction was non-significant.
9	Yamaji et al., 2020 [49]	Systematic review and meta-analysis on decision making of children with cancer	Articles looking at decision making including informed consent, informed assent, shared information. Qualitative research studies which had deliberation features.	Children aged 2 to 18 years who were diagnosed with any form or stage of cancer, and to whom cancer diagnosis was disclosed in high income countries (USA, UK, Netherlands, Canada, Ireland, Switzerland, Finland, Denmark, Sweden)	Four themes emerged that reflected the decision-making process of children with cancer: (a) facing changes brought about by a health threat, (b) preparing for action, (c) asserting one’s choice, and (d) internal and external influences.	• Respecting children’s preferences, values, and emotions may help build trusting relationships and promote their decision-making capability. • Future research should focus on children’s emotions, cognition, development, and interactions with parents and health care professionals.
Primary study articles (*n* = 21)						
10	Aoki et al., 2019 [50, 51]	Randomized Controlled Trial including quantitative Survey	Three steps: 1) Presenting diagnosis and treatment option to the patient, 2) Patient Reflection and Deliberation with health professional, 3) A week later, the patient meets with the nurse to discuss the decision. The nurse’s recommendations are accepted by the patient at the face-to face meeting.	Participants (*n* = 88) aged 20 years or older with a mental health diagnosis from Waseda, Japan	Combined Outcome Measure for Risk Communication and Treatment Decision-making Effectiveness (COMRADE) two subscales: satisfaction with communication and confidence in decision.	• Providing participants with SDM booklet to take-home allowed patients time to reflect on possible options. • Nurse remote support or discussion post the initial diagnostic meeting provided patients with a safe sounding board while exploring available information
11	Sebti et al., 2019 [39]	A focused ethnography, with a participatory approach to promote improvements in sexual health-related knowledge and actions in children/youth	Four key informants (local & cultural experts) in the community provided advisory input throughout the study. (a) local views and understandings of children/youth participation in sexual health-related discussions, decisions, and actions; (b) suitable data collection sites and methods; and (c) relevant key texts.	The study was conducted in Njombe, a Tanzanian rural community. Twenty-eight participants, 8–16 years of age, living in Njombe (Tanzania), were recruited from the HIV youth group.	Four principal themes were identified: (a) knowledge and understanding of sexual health, (b) children/youth value sexual health education and discussion, (c) supports and barriers for participation in sexual health education, and (d) children/youth value participation in their own care and promotion of their health	• The results highlight that young people have the capacities, interests, and values to actively participate in discussions, decisions and actions regarding their health in general and sexual health in particular • The study demonstrates how (a) child/youth agency is socially mediated, as the actions of others can promote or impede the expression of their agency • Community programs can provide effective assistance to support young people’s agency.
12	Barber et al., 2018 [52]	Mixed method feasibility study Qualitative interviews, stakeholder consultations, observations, systematic review, and survey design and implementation	**Stage one:** Systematic review of hypodontia literature. Interviews with and observations of adolescents, parents and dentists **Stage two:** Development of discrete-choice experiment survey **Stage three:** Pre-testing survey with cognitive interviews	Leeds Dentists Institute, UK. Participants were young people aged 12–16 with hypodontia (n = 8), and their parents (n-8) and dentists (*n* = 5).	The discrete-choice experiment survey included 27 attributes on service delivery and treatment outcome.	• In the pre-testing interviews, young people found that the survey difficult to understand and so the survey was modified to increase acceptability. • Pre-testing interviews suggest that the survey encourages decision preference for treatment. • Including young people in the all stages of survey development improved acceptability.
13	Cioana, et al. 2019 [53]	A National Conference to build a national coalition to target the future of adolescent idiopathic scoliosis research and clinical care	The conference combined presentations and roundtable discussions to define an agenda for the coalition. Day 1 involved presentations related to the current landscape of adolescent idiopathic scoliosis research, Day 2 was focused on discussions regarding the development of the coalition.	The conference brought together adolescent idiopathic scoliosis stakeholders including patients, parents, students, clinicians and researchers (*n* = 23) across Canada.	Roundtable discussions were used to identify emerging priority areas to guide the research agenda.	• School, teachers and carers have an important role in promoting decision making for young people. • Transdisciplinary teams to promote priority setting outcomes for youth. • Including youth from the on-set as a critical component to positive outcomes
14	Coad et al., 2008 [54]	Intervention evaluation of setting up a youth council for priority setting	Background literature review Set up the “Youth Council” which used young people friendly interviews and questionnaires on hospital preferences.	Participants were 17 young people aged 12–18 recruited from secondary schools in Coventry, UK. 10 girls and 7 boys, and majority white British ethnicity.	Evaluation workshop after 18 months of running youth council to understand the roles, concerns and satisfactions.	• The youth council advised on thedesign of questionnaires and surveys, developed a Bedside Booklet for the adolescent unit, Trust website, FamilyTalk (communication about conditions with young people), and posters to attract young people in research • This is a long-term commitment which is successful with reward mechanisms, capacity building and creative approaches.
15	Dion et al. 2021 [55]	Qualitative study with peer researchers	Step 1: Working with Peer researchers to identify participant Step 2: Setting up focus groups with peer researchers. Step 3: Converting evidence synthesis to user friendly infographics with peer researchers Step 4:Allow young people to engage with the material. Step 5: Identify their own priority subjects though interactive activities Step 6: Involvement of young mothers to organise the themes	Pregnant and parenting young women under 23 years and their services providers in Canada.	Better Outcomes Registry Network (BORN), focus group Discussions and first -second order themes with contribution from participants	• Important to combine evidence-based synthesis and local context specific knowledge or understanding. • It was important to the young women to choose how their experiences are ordered. • Confidentiality was respected if the young women choose not to publicize their story.
16	Groot et al. 2021 [56]	A qualitative evaluation study consisted of observations of council meetings, in-depth interviews with youth council members, and moderated group discussions.	The collective experiences of pediatric patient engagement from the perspective of adolescents themselves were sought to provide more insights for healthcare providers and researchers on how to involve children in a meaningful way in research and care	The youth council comprised nine members, aged from 12 to 18 years (at the start of the council), five girls and four boys. All of them have (a) chronic respiratory diseases: eight have asthma (ranging from mild to severe) and one has cystic fibrosis from Amsterdam, Netherlands.	Data collection consisted of observations of council meetings, in-depth interviews, and group discussions with the adolescents undertaken by an independent researcher. Topics of the interviews and group-sessions included members’ experiences in the youth council, their motivations (individual and group) and the outcomes	• Young people participating in a youth council, value group engagement, and experience different benefits, from having fun to peer support and feeling more confident. • However, it also shows that long-term and meaningful participation requires an organizational shift that moves from an adult-led agenda towards a youth-led agenda. • There is a need for an organizational climate in which unsolicited advice is valued and facilitated by formal structures to prevent adolescent patients’ frustration and demotivation in the longer term.
17	Guinaudie et al. 2020 [57]	Insights from the co-design and implementation of SDM in knowledge translation, clinical, operational, and research strategies within the ACCESS OM network.	Lived mental health experience is acknowledged and integrated as unique experiential knowledge and unique expertise to advise and to guide the project in collaboration with researchers, mental health clinicians, and decision makers.	ACCESS Open Minds (ACCESS OM) is a patient-oriented research initiative that is transforming and evaluating youth mental health services at more than 16 diverse sites across Canada. ACCESS OM defines ‘patients’ as youth aged 11–25 years, and ‘patient partners’ includes youth patient partners and, family and carers patient partners	ACCESS OM assessed the innovative ways in which SDM strategies might foster effective integrated knowledge translation in a youth mental health research setting.	• SDM strategies foster dialog and partnerships among stakeholders (e.g., youth, family members/carers, clinicians, researchers, and policy makers) by acknowledging that diverse forms of knowledge (specifically experiential, cultural, clinical, and scientific knowledge) can lead to better health and improved social and economic outcomes for patients and communities. • Challenges to SDM include power dynamics, time constraints, project pace, and tokenism.
18	Jordan et al. 2019 [58]	Participatory qualitative interviews were conducted using life grids and pie charts, and transcripts were analysed thematically.	This study explores adolescents’ perceptions and experiences, focusing on identifying the perceived barriers to, and facilitators for, their involvement in shared decision-making by focusing on their lived experiences during consultations.	A sample of nineteen young people between the ages of 13 and 19 years was recruited from young adult (transitional) or paediatric neurology, endocrinology, nephrology or rheumatology clinics in South Wales, UK.	Participatory interviews were conducted to explore the adolescents’ narratives in order to identify possible barriers and facilitators to SDM. Respondents were asked to complete a life grid with important events surrounding their health condition and doctors’ visits. A follow-up semi-structured interview schedule was also derived from the findings of our systematic review	• Significance of the relationship between the HCP and young person, and the importance of reducing the perceived power imbalance. • Nearly all young people indicated a desire for the same or greater involvement in the decision-making process, particularly as they gain more experience with their condition. • HCPs’ behaviour can improve adolescent involvement in SDM by ensuring they speak to patients directly, providing sufficient information about options, inviting questions, and making it clear that they want them to be involved.
19	Lopez-Vargas et al. 2019 [59]	A 1-day workshop.	The workshop process was informed by the James Lind Priority Setting Partnership methodology	Workshop was held in Sydney, Australia, in 2016. Young people aged 11 to 18 years with a chronic condition, parents/caregivers of children aged 0 to 18 years of age with a chronic condition, representatives from health consumer organisations (eg, Cystic Fibrosis Australia, Allergy and Anaphylaxis Australia), health professionals (paediatricians, general practitioners, nurses), allied health professionals (counsellors, dietitians), researchers and policy-makers with expertise or an interest in childhood chronic conditions were eligible to attend the workshop.	The Kaleidoscope Project is an initiative that aims to identify the research priorities for children with a chronic condition. In total, 78 unique research questions were identified and narrowed to the top three research questions identified by each of the seven breakout groups.	• Research priorities focus on education and life participation, psychosocial wellbeing, quality of care and impact on the family. • For young people, there was an emphasis for research to help them maintain a sense of normality and to be empowered for self-management and partnership in care. Specifically, this was expressed in terms of access to educational support, social acceptance and life and community participation.
20	Martinez et al., 2020 [60]	6 week youth led programme	Young people engaged in discussions, multimedia (vidoes) research priorities. Once the priorities were formed, visual storytelling and writing activities to identify health problems, and brainstormed a list of research questions that supported the development of survey form further prioritisation. Young people also used Photovoice to capture images in their neighbourhoods that represent their health priorities.	12 black and Latina young people aged 13–18, a young adult facilitator at the Boston Centres for Youth and Families.	The development of the survey with the young people generated priorities, and the Photovoice analysis.	• The survey deemed the priorities to be mental and sexual health, food access and community safety. • Young people participation fosters deeper empowerment and leadership potential. Policy and health leaders should consider young people in decision about health care.
21	Morton et al., 2017 [61]	Web based Delphi survey to engage young people to prioritise secondary school environmental physical activity interventions	**Stage one:** develop list of potential interventions From a systematic review And secondary data analysis -public advisory panel input and from strategic advisory group input **Stage two:** document preparation for Delphi criteria to include reach, equality, feasibility, effectiveness, acceptability, and cost **Stage three:** Delphi study -round one -feedback to participants -round two -Top 2 interventions selected for feasibility study -feedback to participants	37 stakeholders including young people aged 13–16 years (*n* = 12), parents (*n* = 1), teachers (*n* = 6), health practitioners (7), academics (8) and commissioner (3). Web based study to include diverse stakeholders from different locations.	Web based Delphi survey developed to critique the reach, equality, feasibility, effectiveness, acceptability, and cost of school based physical activity interventions. This was conducted in two rounds, and showed increase consensus with the majority.	• Participants ranked mental health and wellbeing as the most important to consider, followed by enjoying school. • Making the intervention effective was consistently ranked the most important in each round.
22	Pflugeisen et al., 2019 [62]	Formation and first meeting of a community adolescent and young adult oncology council (AYAOC)	Full day workshop for 2.5 hours with non-for-profit CanTeen, young people and researchers. Workshop consisted of open discussion and prioritisation in pairs and small groups. The priorities that emerged were then bought to the big group to rank the top three priorities. Votes were tallied and created a road map for future meetings.	Young people with cancer plus their caregivers, siblings, oncology health professional from Washington State, United States. Hospital settings.	Themes identified from AYAOC discussion included emotional isolation, naivety with and sometimes distrust of the medical system, the lasting impact of cancer on identity, the need for emotionally safe interactions with both individual clinicians and groups of peers, and the desire to take personal action to improve care for future patients	• AYAOC members expressed a drive to share their experiences, advocate for others, and improve health care services for the “next generation” of AYAs diagnosed with cancer. • Sharing stories and connecting with peers may have personal value for individuals. • Channelling the altruistic energy of AYAs and stakeholders into group advisory and advocacy efforts also has value for health care systems, allowing stakeholder insights to inform clinical service delivery and research priorities.
23	Rich et al., 2014 [63]	Evaluation of The Teen Advisory Board Committee through young people, hospital and facilitator perspectives	The aim of the Teen Advisory Committee was to: -Explore adolescent patients and siblings areas of interest -Make recommendations and suggestions to enhance quality and quantity of adolescent patient programs through self -advocacy. -Inform hospital administration, clinicians, and other hospital employees of the work of the committee. -Provide young people with leadership experience, writing proposals, and public speaking	The Teen Advisory Committee, Boston Children’s Hospital began in 2002 with 18 young people ranging in age from 14 to 21 years. 14 were patients, two were healthy siblings of patients, and two were peer leaders from the hospital’s Youth Advisory Program in the Division of Adolescent/Young Adult Medicine. The committee had a teen coordinator and five staff facilitators.	The evaluation on: • Setting and maintaining boundaries • Structured recruitment, application, interviews • One-to-One confidential facilitator/teen meetings • Developmental stage and chronic illness status • -Building a sense of empowerment • Addressing personal overarching concerns	• The program empowers adolescents with chronic illness by leading projects that led to the development of hospital policies, procedures, and quality improvement initiatives.
24	Saunders et al., 2016 [64]	Mixed methods study conducting stakeholder meetings, interviews and survey.	Literature review on community based participatory research to inform stakeholder panels Stakeholder panels with young people, parents and professionals	Stakeholder panel meetings consisted of 11–17 year olds (*n* = 6), parents (*n* = 3) and professionals (*n* = 11). Young people diagnosed with familial hypercholesterolemia (FH), patients with cardiovascular risk due to another chronic illness (e.g., obesity) and patients with no known cardiovascular risks (general population)	Three separate stakeholder meeting were hosted as virtual meetings on webinars To evaluate the stakeholder panel, young people and parents filled in a survey and participated in semi-structured interviews.	• From the stakeholder panel evaluation, they found that there was a power dynamic between stakeholders and researchers. • There are recommendations to share knowledge, build relationship and trust, and improve logistics.
25	Simmons et al., 2009 [65]	Mixed method stakeholder workshop and survey to score priorities.	**Stage one:** sociocultural analysis of populations in New Zealand, Fiju and Tongo in the form of interviews **Stage two:** stakeholder engagement **Stage three:** stakeholder workshop to confirm ANGELO framework using a scoring process (prioritisation) **Stage four:** draft action plan	Stakeholders were recruited from six obesity prevention projects in Australia (n = 3), New Zealand, Fiji and Tonga from 2002 to 2005. Target groups were under-5-year-olds (Australia), 4–12-year-olds (Australia) and 13–18-year-olds.	ANGELO process was developed in the four stages. ANGELO prioritisation elements relate to behaviours, knowledge/skill gaps, and environmental barriers to healthy eating and physical activity prioritised on importance and changeability. This prioritisation creates a list of potential targets for action created in a SMART format.	• The ANGELO framework was found to be flexible and efficient at agreeing an obesity prevention plan with the different communities. • 120 potential behavioural, knowledge, skill and environmental ideas were identified at the each 2-day workshop, leading to prioritisation task. • Adolescents were engaged and took ownership in the process.
26	Simmons et al., 2017 [66]	An uncontrolled cohort study. Development and evaluation of a decision aid.	Web-based decision aid.-	Young people aged 12–25 years (*n* = 66) with varying depression severity. The study was undertaken at two headspace centres that are enhanced primary care services with a focus on youth mental health for young people aged 12–25 years in the northern suburbs of Melbourne, Australia.	Follow ups, where clients were asked if they were able to make decisions before and after decisions aid. Clinicians rated how satisfied they were with decision-making.	• After the decisions aid, young people were more able to make a decision, and had significantly reduced depression scores. • Potential for young person’s collaboration in the decision-making process of their treatment. • Clinicians supported the decision-making choices.
27	Twine et al., 2016 [67]	Community engagement workshops. Delphi prioritisation method, face-to-face discussions and participatory visualisation.	Three workshops between community stakeholders and researchers. - First workshop explained the study, compiled list of priorities and conducted Delphi round one. -Between workshop one and two, the stakeholders received Delphi round two by text message. -Second workshop held face-to-face discussions and conducted Delphi round three. -Between workshop two and three, analysed the consensus and finalised top five priorities. -Third workshop presented a diagram reflecting the relationships between priorities and formulated stakeholder forum.	Thirty two stakeholders from Agincourt HDSS, South Africa, including community Advisory Group, adolescents and parents.	The Delphi approach conducted over three rounds enabled voting of health priorities and reducing the priorities from a list of 10 in the first round to five priorities in the second and third rounds. In the workshops, face-to-face and participatory visualisation facilitated deliberation between stakeholders and researchers.	• There was consensus from stakeholder and researchers that peer pressure and lack of information were underlying causes of adolescent health problems. • The stakeholder forum formed in workshop three worked well with the researchers throughout to further develop public engagement strategies, share preliminary findings, discuss research progress,and identify policy champions to act on research findings.
28	Wysocki et al., 2016 [68]	Mixed methods. Qualitative interviews and web based development of decision aid co-designed by adolescents	Semi-structured interviews with paediatric health care providers were conducted about shared medical decision making. Adolescents and parents were interviewed, mainly separately about retrospective decisions making about adherence to insulin pumps. Interview themes contributed to the design of the web-based decision aid, including insight from a paediatric endocrinologist and a web health expert diabetes nurse.	Health care providers (*n* = 42) including paediatric endocrinologists and certified diabetes educators at 5 paediatric health care sites. Young people with Type one Diabetes, and their parents (*n* = 36). Young people were selected with diverse perspectives of insulin pumps or continuous glucose monitoring, and based in health care setting.	Two web- based decision aids for adolescents and parents, based on their value: 1. Content selection to enable users to access information and navigate freely 2. Repetition of key points 3. Key information in different formats 4. Active learning tactics to engage users with content 5. Maximal information strategies e.g. progress meter 6. Conversation among teens, parents, and clinicians e.g. quiz 7. Text at sixth- to eighth-grade reading level 8. Balanced information 9. diverse age, race, sex, interests, and care choices among teens/families	• Adolescents found quizzes a preferred interactive feature to spur discussions with family and health professionals. • Decision slider recorded thoughts at different time points to track change. • Attention to detail to the design of decision aids can increase the effectiveness of decision making around changes to diabetes for adolescents and parents.
29	Von Scheven et al., 2021 [69]	Focus groups to uncover priority research questions on chronic diseases followed by focus groups to rank the priority questions.	During the first focus group session participants shared their experiences of living (or caring for someone) with a chronic condition and generated an extensive list of questions. The research team then categorized these questions by topic. During the second focus group, participants prioritized the topics they wanted researchers to focus on and ranked the specific questions they were most eager to have answered.	Young people aged between 15 and 23 years and their caregivers from San Francisco, US. Young people with chronic diseases such as Juvenile idiopathic arthritis, migraine headache, Idiopathic pulmonary capillaritis, Type 1 diabetes, Juvenile dermatomyositis, Systemic lupus erythematosus, Inflammatory bowel disease, Psoriasis, Celiac disease, Microscopic polyangitis)	The ranking of the priority topics was compared across the five groups to identify patterns by role and location. Themes included: 1. Health Care System and Care Coordination/Communication2. Insurance/Health Care Coverage; 3. Patient–Parent–Provider Relationship and Communication; 4. Social/Emotional/Family Impact and Support; 5. Transition to Independence: Going from Pediatric to Adult Care. Involvement of Patients and the Public (GRIPP2) survey was also completed.	• Questions posed by young people experiencing different chronic conditions fell under three themes (physical, social–emotional and health care system) and two cross-cutting dimensions (living with a chronic illness and future with a chronic illness). • Use of the Research Prioritization by Affected Communities (RPAC) method, which begins with the patient’s lived experiences provided nuanced insights into the complexity of living with a chronic illness and surfaced under-studied research topics to guide future research investment.
30	Zwaanswijk et al., 2011 [70]	Experimental design to investigate medical decision making by vignettes	Based on a literature review and results of an earlier study, seven vignettes were selected for construction. Six factors were used to construct vignettes: -main subject of consultation -illness stage, prognosis -child age, child emotionality -child’s physical condition -amount of parents’ pre-existing knowledge of the illness	Participants were recruited and took place in three Dutch university-based paediatric oncology centres. Children with cancer aged 8–16 (n = 34), parents (59), and survivors aged 8–16 at diagnosis and currently age 10–30 years (*n* = 51).	Each vignette was followed up with three questions about preferences related to the situations. 1. Information should be given to patient and parents simultaneously (yes/no) 2. The best way to inform the patient 3. The patient should participate in medical decision making (yes/no)	• Health-care providers should repeatedly assess the preferences of both parents’ and patients’ to adapt communication. Healthcare providers’ empathy was important. • Preference for information to be given to child and parents simultaneously.

## Results

**Fig. 2:**
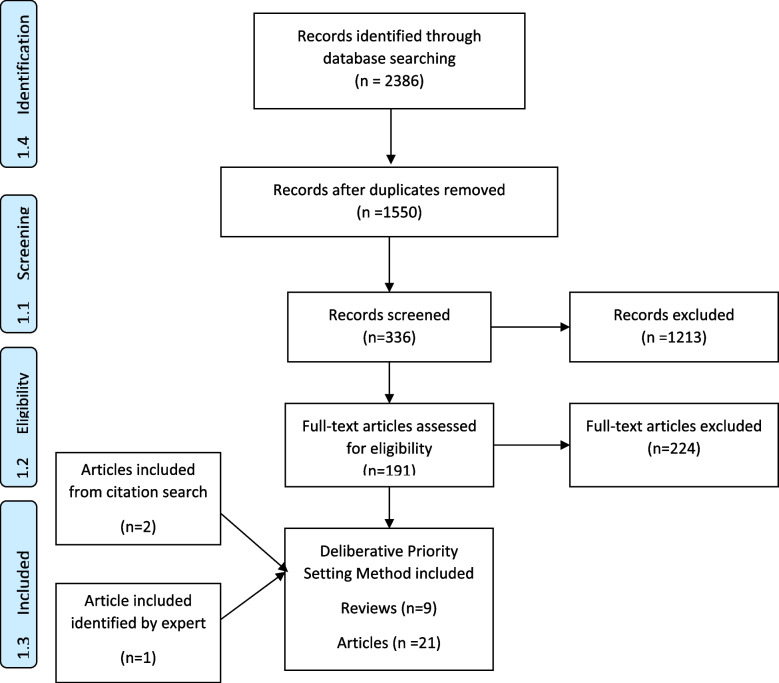
PRISMA flow diagram

A total of 9 reviews and 21 primary studies were identified that used a deliberative priority setting method with young people (Fig. 2). The deliberative priority setting methods were similar in that they all attempted to engage young people in a two-way dialogue with researchers, policymakers, health professionals and parents among others. Table 2 describes the range of deliberative priority setting methods and indicates differences between the methods in the frequency with which and length of time over they engaged young people, the data collection tools that they employed and with whom they engaged.

### Representation

#### Geographic representation

Studies reviewed tended to be conducted in high-income countries (i.e. Australia, Canada, Japan, the Netherlands, United Kingdom, United States), and covered rural and urban areas but this distinction was not clearly reported in most studies. Two additional studies were conducted in rural communities in middle income countries (i.e. South Africa and Tanzania) [39, 71]. One study included participants from three different countries: New Zealand, Fiji and Tonga [65]. The experience of young people from low-and-middle-income countries was under-represented in papers reviewed. Studies tended not to report the different geographical regions within the countries from which young people came. One study held online stakeholder meetings which allowed for inclusion of a wider geographical spread of participants but only those with access to the appropriate technology [64].

#### Demographic representation

Studies included young people of different ages up to 25 years. The majority of studies worked with combined groups of children and young people; some studies grouped together all children and young people under the age of 18. This made it difficult to draw conclusions about the specific experiences of adolescents, which given their developmental stage, are likely to have been significantly different to those of younger children. Very few studies described the ethnic origin of participants [54] and no studies reported the socio-economic status of the young people involved. The major criterion for selection of participants in the studies reviewed was that they were receiving healthcare for a chronic or critical condition. These conditions included mental illness, cancer, obesity, neurological conditions and HIV.

Adults were almost always included in addition to the young people in the decision-making processes described in these studies. This appears to have been necessary to give credibility to the studies’ conclusions. A study in Tanzania involved community groups and local cultural experts in key informant interviews in order to address the local perception that it was not sufficient simply to base decisions on the views of young people [39]. Leaders in this community also provided the research team with their opinion about how best to recruit young people to priority setting deliberations and suggested suitable data collection sites and methods [39]. In a South African study, community leaders, youth organisations and research unit community advisory groups were included in the deliberative process to facilitate the translation of study findings into changes in practice but also to provide a more direct link to policy makers beyond the end of the project [67].

#### Political inclusion

The influence of politics in setting health priorities was rarely discussed in the studies reviewed. One exception was the study in South Africa which involved representation from the African National Congress (ANC) Youth League in recognition of the role that politics and politicians play in setting health priorities for young people [67].

### Structure of the process

#### Structure of deliberative processes and procedures

The studies reported using a range of techniques to engage young people in deliberation about priorities in healthcare and health policy. Five studies created a young people’s committee or council, where young people worked together with stakeholders from hospitals and mental health services, making decisions to enhance young people’s satisfaction, influence hospital strategy, and to empower young people with chronic illnesses [49, 53, 56, 57, 62]. The authors of these studies concluded that health professionals should engage with young people over an extended period of time to build meaningful relationships. They also recommended using incentives and offering training in skills such as advocacy to increase engagement of young people. Three studies facilitated conferences, panels or stakeholder meetings with young people and researchers [53, 54, 63]. Six of the reviews identified described studies that employed methods to improve engagement, including education, coaching, and therapeutic techniques to engage their emotions, prevent emotional isolation and help build trusting relationships [45–47, 49, 57, 72]. One study also included siblings in the ‘Teen Advisory Board Committee’, which was found to deepen and enrich the decision making experience as young people could discuss the decisions with someone they trusted and who was closer in age than a parent [63]. One study that used vignettes to create stories around treatment decision making in a paediatric oncology setting, found that young people and parents preferred to be given information simultaneously [60]. Another used photovoice and storytelling to explore sexual and mental health priorities [39].

#### Digital interventions

Various digital methods were found to be successful in engaging young people in making decisions about their healthcare and health policy. Online decision aids for young people with depression, often used in consultations, created a confidential environment for sensitive issues [60], and also increased accessibility for populations such as those who were physically disabled, lived in remote areas or were without transport. Stakeholder webinars were considered a successful way to engage young people across locations [54]. Younger people found webinars more user friendly than parents, who described accessing the webinars as requiring a ‘generational learning curve’. Different digital media were employed such as video, photographs and a “Decision Slider”, which allowed young people to record their decisions multiple times [45]. Digital methods were also used to recruit young people to deliberations using social media (Facebook) support groups [56]. One research team incorporated face-to-face engagement throughout the deliberation cycle, as past research has found that a combination of face-to-face and digital interactions is most effective [62]. This is, of course, only possible for communities of young people with access to digital technology.

#### Timing of public engagement in decision-making, levels of engagement and opportunities to share and foster mutual respect

Deliberative priority setting methods varied in duration and contact time with young people, ranging from one off meetings to years of engagement, and from face to face to web-based interactions. Studies with organised panels and committees had longer duration and more contact with young people and stakeholders than survey approaches and was felt by young people to be a more respectful and meaningful way to work. Committees sometimes maintained young people’s engagement for months [56, 57, 62]; Coad et al’s “youth council” ran for 18 months [49] and Rich et al. reported that their “Teen Advisory Committee” ran monthly from 2002 until publication in 2014 [53]. Panels tended to maintain contact over a shorter period; the workshop using the ANGELO framework for obesity prevention, for example, which was delivered over 2 days [63], or in 18 webinars [54]. Surveys were more likely to be conducted in a single interaction, the online decision aid for youth depression is one example which was used in a single 50-minute appointment [60]. Similarly, one online Delphi survey was conducted at two time points each consultation lasting 30 minutes [62].

Studies that included young people in all stages of the decision-making and prioritisation process were felt to provide the maximum opportunity and respect for the contributions of young people. This included respecting the anonymity of the young people’s stories [55], giving a young people’s council the opportunity to provide ongoing insights for an acute hospital trust and involving them extensively in the evaluation of impact and in publication of papers [49]. Most studies did not say if young people were given the opportunity to challenge the decision making process, which Abelson considers important as an indicator of mutual respect [40].

Three studies commented on the power imbalance between young people and researchers or parents in the deliberative process [57, 58, 72]; specifically they observed that the research team’s decisions were given more significance than the young people’s. The result of these power imbalances was to systematically exclude young people from the decision-making process, thus disempowering young people from a process that was intended to empower them [60]. To address this, Guinaudie et al. 2020 created separate and shared spaces for young people and family members to consult with each other early in the project and to develop clear messages before contributing to larger multi-stakeholder discussions with research teams [57]. In some studies, young people were given the opportunity to reflect on and feedback about any difficulties they had in making their views heard. In a South African study, young people were given the opportunity to develop strategy for public engagement, research progress and to become champions of the strategies and decisions made following the workshops, communicating how much the researchers valued the young people’s contributions [67]. One study reflected on the power of social desirability and the way young people’s expression was promoted or impeded by the presence of other young people, healthcare professionals, parents and other adults which in turn shapes young people’s decision-making [39]. Studies suggested that future research should focus on developing young people’s capacity for decision making and by respecting and taking into consideration their preferences, values, and emotions [49, 62]. Such development occurs through acknowledging young people’s experience of the interactions they have with healthcare professionals or their parents, the power differential between them and the impact of these interactions in promoting or impeding their agency [39, 49, 57, 58].

### Quality of information given to participants

Studies presented a number of methods to inform selection of priorities to be considered in the process, including literature reviews [41, 46, 54], qualitative data from experts in the field [60, 63, 67] or from young people [45]. It was unclear from these studies whether literature reviews or qualitative data were more useful in informing priority selections [44, 49]. In order to ensure no potential priorities were missed, some studies used both qualitative research and literature reviews to identify lists of options for change to healthcare and health policy for young people [45, 50, 56, 60, 62]. In order to select from these potential priorities, studies described using methods such as decision aids for young people with diabetes [72] and depression [60], discrete-choice experiments for patients with hypodontia [56], and Delphi studies for identifying physical activity priorities [62] and young people’s general health issues in South Africa [67]. Involving young people in the design of these methods improved their acceptability [67]. Decision aids and shared decision-making toolkits aided the delivery of high-quality information to young people, health professionals and parents, which decreased decision conflict and positively impacted on the treatment adherence for young people with chronic conditions [47, 51].

Visual methods were useful in aiding young people’s decision-making. One study used vignettes with pictures and stories, which were found to be especially effective for communicating about sensitive issues such as childhood cancers [60]. Jordan et al. (2019) used ‘life grids’ to explore important events in the lives of young people with long term health conditions and their interactions with doctors, and then used pie charts to explore discrepancies between the role that the young people wanted in healthcare decisions and how interactions with doctors affected their ability to take part in decision-making [58].

### Outcomes and decisions

Abelson identifies three important types of outcome against which the deliberative process can be evaluated: 1) achievement of consensus, 2) participant satisfaction, and 3) legitimacy and accountability [40].

#### Achievement of consensus

Reaching consensus was an important goal for all types of deliberative processes whatever route to decision making was taken because it was required to deliver decisions on the healthcare and health policy priorities to be addressed [54, 63–65]. Some studies actively managed the process of reaching consensus through use of Delphi methods, where a panel of experts is asked in a series of consultations to select from a pre-defined list of priorities, their responses are aggregated and shared after each round until consensus is reached [67, 73]. Lopez-Vargas et al. 2019, who conducted a one-day workshop with children with a chronic condition asked each participant to generate a research questions that was important to them, and each question, of which there were 78, was then ranked in terms of importance by the group, which generated the top three questions to be explored in more depth [59]. Surveys of other types were also used to reduce decision conflict and generate consensus [52, 61, 66].

#### Participant satisfaction

Deliberative processes that were more genuinely inclusive of their opinions were felt by young people to be more satisfactory [39]. Studies which generated greater participant satisfaction were those of longer duration and more frequent contact [54, 63], panel discussions which gave young people confidence to speak out [65] and skills in leadership [60, 62], and those that included young people in all stages of the priority setting process including designing the decision making tool [52, 53]. Participant satisfaction was also greater when they perceived that the decisions they made were for wider benefit and provided better health outcomes for others than themselves [62]. One systematic review and meta-analysis found that, although shared decision making techniques such as workshops, information sheets, videos and websites significantly improved knowledge of disease and reduced decision conflict, they did not improve patient satisfaction [48].

#### Legitimacy and accountability

Studies were more or less legitimate depending on the degree to which young people’s input was incorporated into final decisions made by health care managers and policy makers. One positive example of this were two studies where a hospital trust reported on the outputs of young people’s committees which included advisory roles and creating patient information and explained the direct impact of these outputs on hospital strategy [54, 63]. Three reviews identified found that some studies excluded young people from the final decision-making process, and the young people’s capacity to prioritise was largely ignored [42, 43, 48]. Contrastingly, three studies encouraged young people to make final decisions on health-related matters; these were focused on hospital strategy, mental health and obesity prevention [54, 65, 66]. Issues with the power dynamic between researchers, parents and young people were also reported in some studies [54]. Legitimate participation of young people in decision making clearly requires a move from an adult-led agenda towards a youth-led agenda [56].

Accountability in terms of how young people’s input into decision making influenced outcomes was rarely discussed. An unusual example is described in a study in South Africa where a community forum was held following the priority setting process, which aimed to communicate to community leaders and disseminate widely young people’s health priorities [67]. Despite most studies reviewed suggesting that involving young people in decision making could improve intervention design and add value to healthcare systems, none described how this process influenced healthcare delivery.

## Discussion

This scoping review identified 9 reviews and 21 studies that described the use of deliberative priority setting techniques in engaging young people with healthcare and health policy decisions. Studies in this review were mainly conducted in high-income countries, included young people and adults together in shared decision-making and rarely reported participants’ demographic status.

### Which deliberative methods have been used to engage young people in healthcare and health policy priority setting?

A number of different deliberative priority setting methods have been specifically developed for use with young people including the James Lind priority setting tool and ANGELO framework, setting priorities with committees, councils, community groups and conference panels, using surveys such as decision aids, Delphi methods, and creative methods like vignettes, photovoice and life grids. The deliberative priority setting methods were similar in that they all attempted to engage young people in a two-way dialog with researchers, policymakers, health professionals and parents among others.

Very few of the studies describing development of deliberative priority setting methods for young people make reference to the priority setting methods that are frequently used with adults [17]. Such methods used with adults include Dialogue methods (Netherlands) [16], Deep Inclusion (United States) [17], Choosing All Together (United States) [18], and Global Evidence Mapping (Australia and New Zealand) [27]. One exception of a priority setting method that was developed for adults but has been used with young people is the James Lind priority setting tool [14]. Manafo et al. (2018), however, described all this list of methods developed for adults to be inclusive, objective, and specific to the priorities of stakeholders involved [17]. It follows that there may be features of these methods that could successfully engage young people in developing a sense of autonomy and perceived control over their health, facilitating their capacity to be effective adult health users [9–11].

### What features of these methods make them effective in engaging young people with healthcare and health policy priority setting?

Abelson’s criteria were used for evaluating deliberative processes and their success in engaging young people [40]. Respectful and inclusive priority setting processes were those that involved young people in all stages of the process, including in designing the study, worked with young people for an extended period of at least a year, used digital methods together with face to face to determine priorities and took steps to address power differentials from the start of the project. Combining evidence from literature reviews with qualitative exploration with young people, parents and researchers was found to generate the fullest list of priorities for presentation to the young people and other stakeholders. Delphi methods were seen as particularly useful tools for ranking priorities and creative methods such as using vignettes improved young people’s understanding of topics under consideration. All studies reported that consensus was reached between participants, but the extent to which the young people made the final decisions, and therefore the legitimacy of the studies, was greater in some studies than in others.

The deliberative priority setting processes varied from a single 30–60-minute commitment from the young people, to meeting monthly over many years. Ongoing contact with young people is described as one of the key principles of the engagement process [74]. There are specific issues with involving young people in the long term that need to be considered. This was highlighted in one study that found challenges with scheduling webinars as young people transitioned over study courses or between high-school, college or full time employment [64], emphasising the need to monitor changes in circumstance and commitments of the young people if their involvement is to be sustained. Providing appropriate training and support is also important for maintaining young people’s commitment [75]. These authors identified that engaging young people over time strengthened the trust and respect between young people, health professionals and researchers, and increased young people’ ability to make a meaningful contribution. Issues of funding and sustaining young people’s long-term involvement were somewhat surprisingly not raised by these studies. Somewhat surprisingly young people appeared to have no issues with the quality of information provided to support the priority setting activities. Fishkin’s work on deliberative democracy suggests that this is a key factor in the success of deliberative processes [76]. One of the ways in which studies addressed this issue was to train young people to facilitate youth councils [49, 63]. These young facilitators were seen as credible sources of information for other young people involved in the process.

The undermining effects of power imbalances between different types of participants on young people’s autonomy was raised in multiple studies [39, 49, 57, 58, 64, 72]. One study took a proactive approach at the beginning of the project to reduce the power imbalances by creating neutral spaces in which all participants views were considered equally [57]. Exemplar studies in this paper have shown that shared decision making with young people is beneficial to building their skillset, agency and confidence, particularly important for young people as they transition towards adulthood.

The review also identified deliberative priority setting methods that may not work so well for all young people. For example, digital means of engagement may not work as well for young people in low resource settings and in the global south where access to internet connections and reliable digital technology is limited. It is also clear that it should not be assumed that this excludes all forms of digital communications; use of platforms such as WhatsApp has been shown to work surprisingly well as part of interventions to improve adolescent health in South Africa [77]. Digital communications may, however, need to be adapted if they are to be inclusive of young people with sensory needs such as speech and sight challenges. Specific work needs to be carried out with these young people to develop deliberative priority setting processes that work for them. Other issues were identified in using deliberative processes with young people. Short timeframes for involvement, for example, carrying-out a Delphi survey over two 30-minute periods [62] and a single 50-minute appointment [60], gave less opportunity for young people to build relationships with peers and adults and less chance to build their decision-making capabilities. Although this particular instance reflected the constraints of the research scope, funding and capacity, it does suggest that reaching a successful conclusion of these processes for young people takes time.

### Strengths and weaknesses

A strength of this scoping review is that it was able to identify diverse deliberative techniques, and was able to give more breadth of the available literature and depth in terms of the amount of information, compared to a systematic review [37]. Our initial search in 2019 found only 13 studies up until 2019, yet when we updated the search in 2021, we found 17 more studies between 2019 and 2021. This suggests that there has been an increase in the use of priority setting with young people within this time. The update of the review is a strength to this paper. Two reviewers simultaneously screened the papers however, only one researcher screened 100% of the papers. Another five researchers divided up the task and screened 10%. A limitation of this study is that it was difficult to identify one method as being the most effective in increasing engagement with young people in priority setting. Instead, different features of the techniques were considered successful (see recommendations in Table 3 below). Abelson et al’s approach to evaluating deliberative priority setting was important to structure the narrative synthesis to identify the key features in the papers [40]. Abelson’s evaluation criteria were limited however, in how far they are able to specify the extent to which criteria have been met.

**Table 3:** Ten recommendations to engage young people in setting priorities

1. Include young people in all stages of the deliberative priority setting process;	
2. Use a mixed method approach to select information including a review of the literature, and qualitative insight from experts and young people;	
3. Long-term commitment and frequent contact with young people to build trust, respect and to increase their decision-making capacity;	
4. Develop online priority setting tools as, young people increasingly value digital engagement;	
5. Build in training opportunities for young people to develop their leadership and advocacy skills;	
6. Plan how to manage potential unwanted power dynamics with young people from the beginning of the project;	
7. Involve community leaders, parents and siblings in the priority setting process to improve satisfaction;	
8. Evaluate the priority setting method to identify challenges with decision-making;	
9. Value the time investment of young people and ensure priority setting methods respect their changing school, work and social commitments;	
10. Conduct cost-effective analysis to strengthen the value of deliberative approaches.	

### Implications

The WHO–UNICEF–Lancet Commission calls for young people to be involved in creating policy that concerns them and their health [3, 4]. Efforts to involve young people in policy and decision-making and in research are currently hampered by a lack of awareness of methods for engaging them effectively [78]. This review suggests that genuinely deliberative methods involving dialogue and mutual respect between young people, parents and professionals which go further than consultation not only achieve consensus on priorities for action but are also the most satisfying and beneficial for participants. It is widely held that involving young people in healthcare and health policy-making will also produce services with which they want to engage. Findings from this review may also be useful to policy makers who seek ways to collaborate with a diverse group of young people in producing health policy in a way that reduces power imbalances.

Deliberative priority setting processes are also thought to increase health equity and literacy young people the opportunity to build their skills, knowledge, understanding and gain confidence in their ability to control over their own lives [79]. The WHO report on engagement and participation for health equity acknowledges that involvement of those with the least heard voices including young people, can lead to more equitable public health policies [79]. Deliberative priority setting processes may not directly tackle economic inequalities and social determinants of the health of young people but they do involve in activities that improve access to and experience of services. A by-product of deliberative processes seems to be that young people may develop more meaningful relationships with parents, carers and peers, argued by the Association of Young People’s Health to improve both their physical and mental health [12]. There is, in addition, growing evidence that meaningful participation of young people in decision-making promotes social cohesion, creates more equal communities and helps adolescents make better informed and more empowered transitions to adulthood [7]. Its notable that none of the studies covered by this review addressed the cost-effectiveness of these deliberative priority setting with young people. Information on cost-effectiveness would assist policy makers in deciding whether or not to adopt these types of approaches with young people. From this review, we have 10 recommendations (Table 3).

## Conclusion

This review does not provide definitive answers to questions about the most effective way of engaging young people in deliberative priority setting. A range of features was identified, however, which appeared to be associated with high participant satisfaction, achieving genuine consensus and were felt to generate outcomes which genuinely reflected participants’ priorities. The WHO, UNICEF and other global organisations wishing to engage young people in setting priorities for healthcare and health policy might benefit from designing their interactions with young people around these key features of deliberative priority setting processes.

## Supplementary Information

**Additional file 1.**

## Data Availability

Published secondary data.
