# Supplementary material for: How do we best engage young people in decision-making about their health? A scoping review of deliberative priority setting methods
Source: Int J Equity Health. 2023 Jan 25;22:17. doi: 10.1186/s12939-022-01794-2 (PMC9876416; doi:10.1186/s12939-022-01794-2)
Supplement: Supplementary file 1 — Additional file 1. [file 12939_2022_1794_MOESM1_ESM.docx]

Appendix A

Data collection form

Priority Setting in Adolescent’s Health

# Inclusion criteria

| **Publication type** | **Published journal articles** |
| --- | --- |
| **Study Design** | **Interventions** |
| **Population** | **Adolescents** |
| **Interventions** | **Priority setting interventions** |
| **Outcomes** | **Health** |
| Population | (adolescent* OR youth OR young OR teen* OR student* OR girl* OR boy* OR pupil* OR puberty* ) |
| Intervention | (“priority setting” OR “resource allocation” OR “decision setting” OR “decision making” OR “decision exercise” OR prioritization OR “economic evaluation” OR “research priority” OR “consensus build*” OR “research consult*”)  AND  (“patient engagement” OR “patient participation” OR “patient consultation” OR “patient involvement”)  OR  “public engagement” OR “public participation” OR “public consultation” OR “public involvement”)  OR  “stakeholder engagement” OR “stakeholder participation” OR “stakeholder consultation” OR “stakeholder involvement” |
| Outcome | Health OR health care OR healthcare |

# Study eligibility

| Study Characteristics | Inclusion criteria: | | Exclusion criteria: |
| --- | --- | --- | --- |
|  |  |  |  |
| Type of study | Intervention, primary data, systematic reviews | | Protocols, commentary articles, conference papers, abstracts |
| Participants | Adolescents 11-35 years  Including marginalised groups and all countries | | Children under 11 years  Adults above 35 years  Parents/carers of adolescents  Only health providers of adolescents  Students |
| Types of intervention | Deliberative priority setting (tools or methodology)  “(1)Research planning included gathering  and analysing identified research priorities by engaging patients and the public along with clinicians and researchers,  (2)followed by prioritization of topics through dialogue between all stakeholders”  Examples: Choosing Healthplans All Together (CHAT) tool and James Lind Alliance priority setting partnership. | | Patient or stakeholder engagement without priority setting  Consultative priority setting (solely focus groups, interviews, meetings, surveys) |
| Types of comparison |  | |  |
| Types of outcome measures | Health | |  |
| INCLUDE | | EXCLUDE | |
| Reason for exclusion |  | | |
| Notes: | | | |

**DO NOT PROCEED IF STUDY EXCLUDED FROM REVIEW**

.
